# 

**Published:** 1827-08

**Authors:** 


### MONTHLY LIST OF MEDICAL BOOKS.

Transactions of the Medical and Physical Society of Calcutta. Vol. II.—Calcutta, 1826.

Oratio Harveiana prima in Novis Ædibus Collegii habita Sext. Kalend. Jul. An. MDCCCXXVI. A Pelham Warren, M.D. &c.—Londini, 1827.

Lectures on the Operative Surgery of the Eye; or, an Historical and Critical Inquiry into the Methods recommended for the Cure of Cataract, for the Formation of an Artificial Pupil, &c. &c. &c. By G. J. GUTHRIE, F.R.S. &c. Second Edition, with seven explanatory Plates.—London, 1827.

A Treatise on Gun-Shot Wounds, on Inflammation, Erysipelas, and Mortification, on Injuries of Nerves, and on Wounds of the Extremities requiring the different Operations of Amputation, &c. By G. J. GUTHRIE, F.R.S. &c. Third Edition, with five explanatory Plates.—London, 1827.

An Inaugural Discourse, delivered at the Opening of Rutgers Medical College, in the City of New York, on Monday, the 6th day of November, 1826. By DAVID HOSACK, M.D. F.R.S. &c.—New York, 1826.

Observations on the Medical Character. Addressed to the Graduates of the College of Physicians and Surgeons of New York, at the Commencement, held on the 4th of April, 1826. By D. HOSACK, M.D.—New York, 1826.

Medical Botany, No. VII.; containing Rheum Palmatum, Tormentilla Erecta, Aconitum Napellus, and Iris Florentina.—Of these we need only say, that in execution they are equal to those which have preceded them.

### METEOROLOGICAL JOURNAL,

### NOTICES.

*The letter of* M. D. *cannot be inserted. Communications of such a kind ought always to have the name of the writer.*

*Papers have been received from* Dr. CHAMBERS, Dr. LEE, Mr. J. GUTHRIE, Mr. CURTIS, *and* Mr. TEEVAN, *which, with various others, came to hand late in the month, and have necessarily been postponed.*

Mr. C. WILLIAM'S *note was mislaid, which gave rise to the unintentional neglect of his request.*

